# MRI appearance of chronic subdural hematoma

**DOI:** 10.3389/fneur.2022.872664

**Published:** 2022-08-08

**Authors:** Dimah Hasan, Omid Nikoubashman, Rastislav Pjontek, Andrea Stockero, Hussam Aldin Hamou, Martin Wiesmann

**Affiliations:** ^1^Department of Diagnostic and Interventional Neuroradiology, University Hospital Rheinisch-Westfälische Technische Hochschule (RWTH) Aachen, Aachen, Germany; ^2^Department of Neurosurgery, University Hospital Rheinisch-Westfälische Technische Hochschule (RWTH) Aachen, Aachen, Germany

**Keywords:** hematoma, subdural, chronic, magnetic resonance imaging, empyema

## Abstract

**Objective:**

We aimed to describe the magnetic resonance imaging (MRI) characteristics of chronic subdural hematoma (cSDH) and to ascribe MRI patterns.

**Methods:**

A total of 20 patients having 27 subdural hematomas underwent contrast-enhanced (CE) MRI of the brain at our institution between April 2019 and May 2021. The images were independently evaluated by two experienced neuroradiologists with regard to imaging characteristics on T1w, T2w, T2^*^-GRE, FLAIR, diffusion-weighted magnetic resonance imaging (DWI), and CE images.

**Results:**

The signal characteristics of cSDH on T1- and T2-weighted images were rather heterogeneous. The majority of hematomas (74%) had internal septations. Surprisingly, contrast enhancement along the outer membrane adjacent to the cranium was noticed in all hematomas. There was also contrast enhancement along the inner membrane adjacent to the brain in more than one-third of the hematomas (37%). In approximately two-thirds of the cSDH (62%), there was a mass-like enhancement of the hematoma. Most hematomas (89%) were partially hypointense on T2^*^-GRE and/or susceptibility-weighted imaging (SWI). Restricted diffusion was detected in approximately one-third of the hematomas (33%).

**Conclusion:**

Consistent contrast enhancement along the outer membrane, triangular-shaped contrast enhancement at the borders of the cSDH, and infrequent enhancement of the inner membrane may help to distinguish cSDH from other entities such as empyema and tumors. Mass-like enhancement may refer to non-solid hematomas and could be an indicator for hematoma growth and a possible surrogate for successful endovascular embolization. Restricted diffusion in a subdural mass is not specific for empyema but may also be found in cSDH.

## Introduction

Chronic subdural hematoma (cSDH) is a common intracranial hemorrhage, which affects mainly the elderly and is usually caused by trauma (1). It is one of the most common conditions in the neurological disciplines (1). cSDH is usually diagnosed *via* non-contrast computed tomography (CT), which is the most common imaging modality due to its sensitivity, widespread availability, and rapid acquisition time (2, 3). In contrast, magnetic resonance imaging (MRI) has had a limited role in evaluating subdural hematomas despite the fact that prior studies showed the superiority of MRI in the detection of SDH and the evaluation of the surrounding meninges, especially when contrast-enhanced (CE) MRI is used (4–6). Data in the literature about MRI aspects of cSDH are nevertheless very scarce and either focus on T1 and T2 contrasts or originate from case reports and smaller case series. However, in the last two decades, MRI has been immensely developing with regard to imaging resolution, functional capacities, and acquisition time (7). Hence, MRI could deliver important information about cSDH in a cost and time-efficient manner, and without exposure to radiation. It could also have the ability to better characterize the hematomas, for instance, with regard to internal septa, which could be important in steering the surgical decisions in this group of patients, especially in treatment-resistant cases (6, 8–10). MRI may also be superior to differentiate cSDH from other pathology in the subdural space, of which empyema is the most important differential diagnosis (11). Restricted diffusion and contrast enhancement are considered indicative for subdural empyema (12, 13). However, there is currently no data available on how frequently these imaging characteristics can be found in cSDH.

Hence, the aim of this study was to describe the characteristics of cSDH on CE MRI and to ascribe MRI patterns.

## Methods

### Patients

The protocol for this retrospective study was approved by our local ethics committee. As this was a retrospective study, written informed consent was not required from patients. For our analysis, we searched our imaging database for patients with cSDH who received CE MRI scans between April 2019 and May 2021. Subdural hematomas were defined as chronic if the documented traumatic event occurred more than 3 weeks before admission. When no history of trauma was given, an SDH was defined as chronic if its density was hypoattenuated to brain parenchyma on the initial CT scans. We excluded patients who underwent neurosurgery before MRI and patients with surgically proven empyema. This left 20 patients to be included in our study. Seven of these 20 patients (26%) had bilateral hematomas, which results in a total of 27 analyzed hematomas in our study.

All 27 hematomas were surgically drained. Material for laboratory analysis including microbiological examination was obtained in 20 of 27 cases. The diagnosis of cSDH was confirmed by surgical evaluation in all cases. None of the hematomas showed signs of infection in the laboratory analyses.

### Imaging protocol

All examinations were performed on a 3 Tesla MRI system (MAGNETOM Prisma, Siemens Healthcare, Erlangen, Germany) with a standard 20-channel head coil. All patients received T2-weighted FLAIR (CSF- and fat-signal-saturated T2 Turbo spin-echo inversion recovery sequence; TR = 9,000; TE = 98 ms), T1-weighted imaging (T1w, motion-artifact reduced Turbo inversion recovery magnitude sequence: T1 TIRM Blade dark fluid; TR = 2,300; TE = 34 ms), GRE T2^*^-weighted imaging (T2^*^w, gradient echo sequence; TR = 1,100; TE = 19.9 ms), and diffusion-weighted magnetic resonance imaging [DWI; b = 1,000, spin-echo echo-planar sequence (SE-EPI)] with apparent diffusion coefficient (ADC) in transversal orientation with 3 mm thickness. All patients received T2-weighted imaging in sagittal orientation (T2w, Turbo spin-echo sequence; TR = 4,800, TE = 94 ms). In addition, 13 patients (18 hematomas) received an additional transversal susceptibility-weighted imaging (SWI; TR = 27, TE = 20 ms) and 14 patients (20 hematomas) received also high-resolution (0.6 mm) and strongly T2-weighted three-dimensional constructive interference in steady-state imaging (CISS, gradient echo sequence; TR = 8.2, TE = 3.72 ms) focused on the hematoma. After application of weight-adapted intravenous gadolinium-based contrast media (Gadovist^®^ 1.0 mmol/ml, Bayer Vital GmbH, Leverkusen, Germany), T1-weighted images in transversal and coronal orientation were obtained in all patients (T1-TIRM Blade dark fluid; TR = 2,300 and TE = 34 ms). Fifteen patients (21 hematomas) received CE T2-weighted FLAIR imaging in transversal orientation.

A total of 19 patients (25 hematomas) received non-contrast CT scans on the same day or up to 3 days before MRI.

### Radiological assessment

Two experienced neuroradiologists reviewed all imaging data with regard to MRI signal intensities, occurrence of diffusion restriction, presence of internal septa, presence of susceptibility artifacts as an indication of hemosiderin deposition, and the pattern of contrast enhancement. The signal intensities in T1- and T2-weighted images were described as hyperintense or hypointense in comparison with the cerebral cortex.

The width of hematomas was determined on MRI and defined as maximal diameter in coronal orientation perpendicular to the skull curvature. The thickness of the inner membrane was measured on CISS images. The imaging characteristics of hematomas on CT, if available, were also reviewed and defined as hypodense, hyperdense, and isodense in comparison with cerebral parenchyma.

### Statistics

Results are given as median with interquartile range (IQR) unless specified otherwise. All numbers are indicated hematoma-wise (*n* = 27) unless specified otherwise.

## Results

### Baseline characteristics

We included 20 patients with a median age of 76 years (IQR: 66–84). In 9 patients (45%), there was a history of trauma weeks to months before admission. The remaining 11 patients had no history of trauma. Seven patients received an antithrombotic therapy with anticoagulants or antiplatelet medication. Two patients had hematologic cancer and one patient had intracranial cerebrospinal fluid (CSF) hypotension syndrome as predisposing factors for the subdural hematomas.

The total number of analyzed hematomas in our study was 27. Thirteen hematomas (48%) were on the right side and 14 on the left side. The median hematoma width measured on MRI was 21 mm (IQR: 16–27 mm).

### Imaging characteristics

**Figure 1:**
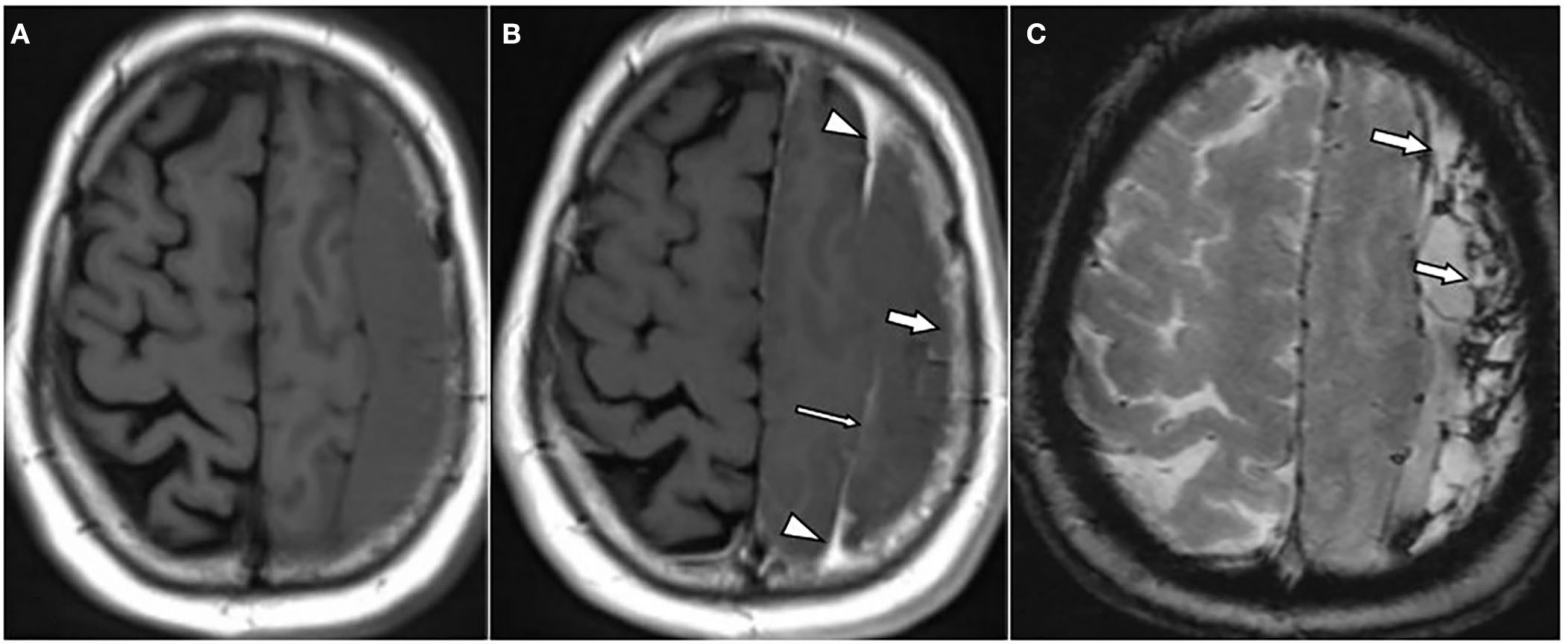
A 62-year-old male patient with chronic subdural hematoma (cSDH) over the left convexity. Axial T1w images pre **(A)** and post **(B)** contrast administration as well as an axial T2*w gradient echo image **(C)**. There is enhancement of the inner [**(B)**: thin arrow] and outer membrane [**(B)**: arrow] as well as a roughly triangular, spandrel-like contrast enhancement at the borders of the hematoma [**(B)**: arrowheads]. On T2*-GRE **(C)**, there is hemosiderin staining adjacent to the dural surface and septa [**(C)**: arrows].

**Figure 2:**
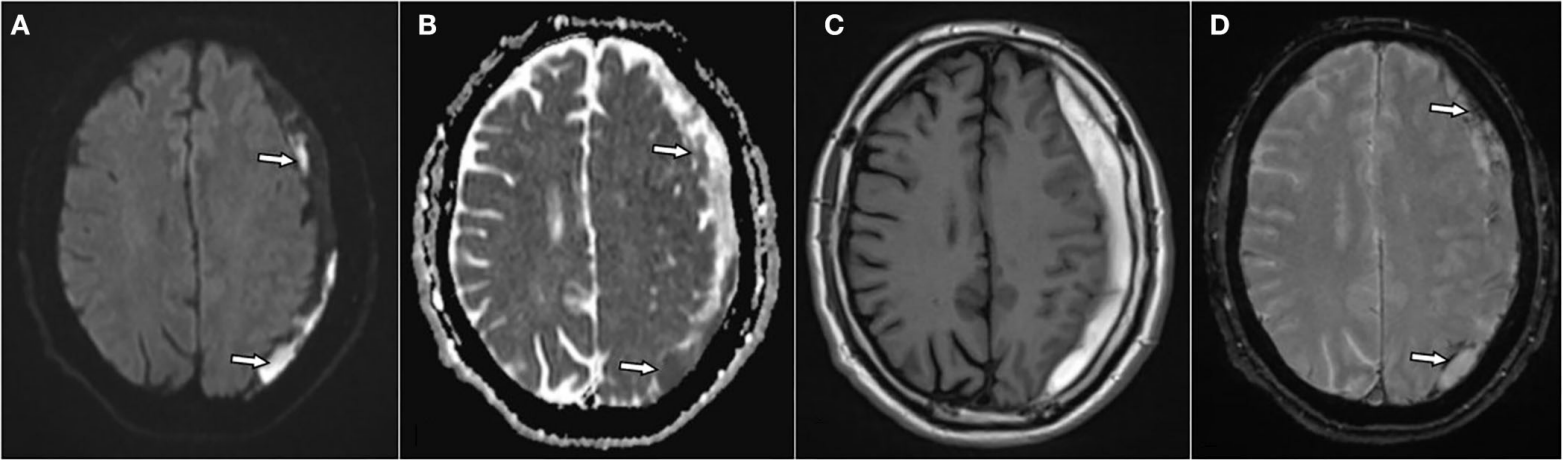
A 66-year-old male patient with chronic subdural hematoma (cSDH) over the left convexity. Axial diffusion-weighted image (DWI) **(A)** and respective apparent diffusion coefficient (ADC) map **(B)**, T1w image **(C)**, and T2* gradient echo-weighted image **(D)**. There are patchy areas of diffusion restriction within the hematoma [**(A,B)**: arrows]. The hematoma is homogenously hyperintense on T1w images **(C)** and shows hemosiderin staining adjacent to the inner membrane and septa [**(D)**: arrows] on T2*-GRE.

**Figure 3:**
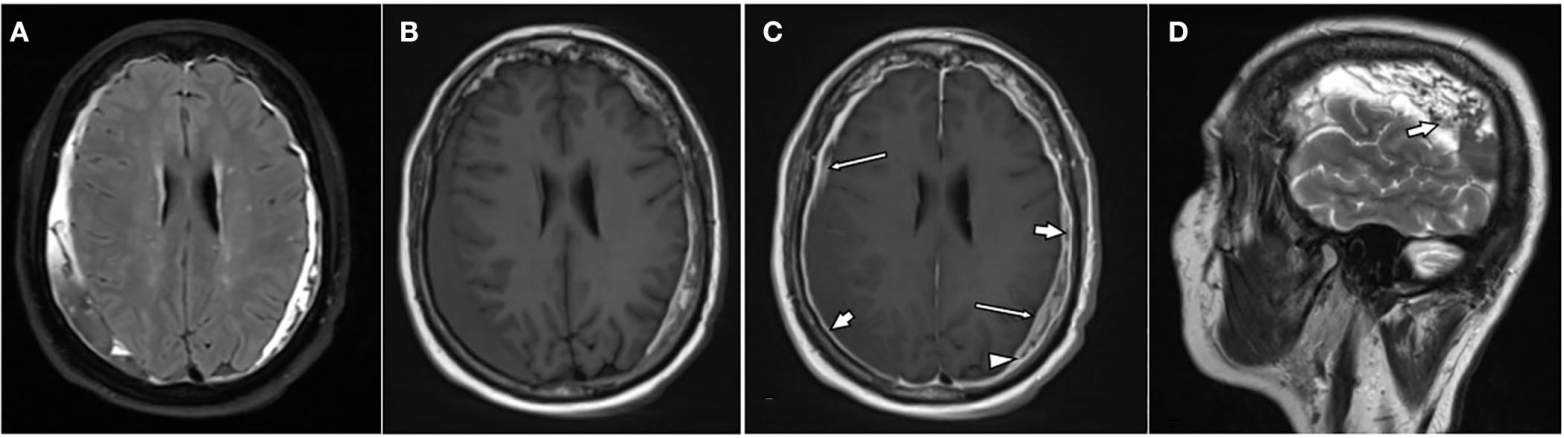
A 76-year-old female patient with bilateral chronic subdural hematomas (cSDHs). Axial-T2-FLAIR image **(A)**, axial T1w images pre **(B)**, post **(C)**, and contrast administration as well as a sagittal T2w over the right hematoma **(D)**. The hematomas are of mixed signal intensity on T2-FLAIR **(A)**, T1w **(B)**, as well as T2w images [**(D)**: arrow]. There is enhancement of the inner [**(C)**: thin arrows] and outer membrane [**(C)**: arrows] as well as spandrel-like contrast enhancement at the borders of the hematoma [**(C)**: arrowhead].

Magnetic resonance imaging signal characteristics and CT imaging characteristics are summarized in Tables 1, 2, respectively. On T1- and T2-weighted images, we observed rather heterogeneous signal intensities (Figures 1–**3**). On T2w, hematomas were hyperintense in 41% of the cases (11 hematomas) and showed mixed signals in the other 59% of hematomas. In contrast, there was more variation in T1w images with 7 hypointense hematomas (26%), 4 hyperintense hematomas (15%), and 16 hematomas with mixed signals (59%).

**Table 1:** Summary of MRI characteristics of chronic subdural hematomas (cSDHs).

	**Signal characteristics on T1w images**	**Signal characteristics on T2w images**	**Signal characteristics on T2w FLAIR images**	**Signal characteristics on GRE images**	**Septations**	**CE outer membrane**	**CE inner membrane**	**CE mass-like**
Number of hematomas, *n* (%)	• Hypointense = 7 (26%) • Hyperintense = 4 (15%) • Mixed intensity = 16 (59%)	• Hyperintense = 11 (41%) • Mixed intensity = 16 (59%)	• Hypointense = 1 (4%) • Hyperintense = 10 (37%) • Mixed intensity = 16 (59%)	• Mass-like hypointensity = 14 (52%) • Linear hypointensity = 10 (37%)	20 (74%)	27 (100%)	10 (37%)	17 (62%)

**Table 2:** MRI and CT characteristics of chronic subdural hematomas (cSDHs).

**Hematoma No**.	**Density on CT images**	**Signal characteristics on T1w images**	**Signal characteristics on T2w images**	**Signal characteristics on T2w FLAIR images**	**Signal characteristics on GRE images**	**Septations**	**CE outer membrane**	**CE inner membrane**	**CE mass-like**
1	Isodense	Homogeneously ↓	↓ and ↑ with layers	↓ and ↑ with layers	Mass-like ↓	0	+	+	0
2	Mixed density	Homogeneously ↓ with ↓ septations	Homogeneously ↑ with ↔ to slightly ↑ septations	Homogeneously ↑ with ↓ septations	Linear ↓	+	+	+	0
3	Hypodense	Slightly ↓ with patchy ↑ areas	Homogeneously ↑ with ↓ septations	Homogeneously ↑ with ↓ septations	Linear ↓	+	+	0	+
4	Mixed density	Mostly ↔ to ↓ in apical layers and ↑ in basal layers	Mostly ↑ with severely ↓ parts and ↓ septations	Mostly ↑ with severely ↓ parts and ↓ septations	Mass-like ↓	+	+	+	+
5	Mixed density	Homogeneously ↔ with only small ↑ spot	Mostly ↓ with layers and septations	Mostly ↓ with layers and septations	Mass-like ↓	+	+	+	+
6	Mixed density	Mostly ↑ with patchy ↓ parts	Mostly ↑ with severely ↓ layers and ↓ septations	Mostly ↑ with severely ↓ layers and ↓ septations	Mass-like ↓	+	+	+	+
7	Mixed density	Slightly ↓ rostral, mostly ↑ occipital	Mostly ↑ with ↓ septations and ↓ parts occipital	Mostly ↑ with ↓ septations and ↓ parts occipital	Linear ↓	+	+	0	0
8	Mixed density	↓ and only slightly ↑ area	↓ and ↑ with layers and septations	↓ and ↑ with layers and septations	Mass-like ↓	+	+	+	+
9	–	Heterogeneous (slightly to severely ↑ with small areas ↔ to slightly ↓) with layers and septations	Mostly ↑ with ↓ layers and ↓ septations	Mostly ↑ with ↓ layers and ↓ septations	Mass-like ↓	+	+	0	0
10	–	Heterogeneous (slightly to severely ↑ with small ↔ to slightly ↓ areas) with layers and septations	Mostly ↑ with ↓ layers and ↓ septations	Mostly ↑ with ↓ layers and ↓ septations	Linear ↓	+	+	0	0
11	Hypodense	Homogeneously ↓	Homogeneously ↑	Homogeneously ↑	Linear ↓	0	+	0	+
12	Hypodense	Homogeneously ↓	Homogeneously ↑	Homogeneously ↑	Linear ↓	0	+	0	+
13	Hypodense	Variously ↑ with ↓ septations	↑ with ↓ septations	↑ with ↓ septations	Linear ↓	+	+	0	+
14	Mixed density	Homogeneously ↓	Homogeneously ↑	Homogeneously↑	Linear ↓	0	+	0	+
15	Hypodense	Homogeneously ↓	Homogeneously ↑	Homogeneously ↓	0	0	+	0	+
16	Hypodense	Homogeneously ↓	Homogeneously ↑	Homogeneously slightly ↑	0	0	+	0	+
17	Mixed density	Mostly heterogeneous ↑ with small patchy ↓ parts	↓ and ↑ with layers and septations	↓ and ↑ with layers and septations	Mass-like ↓	+	+	0	+
18	Mixed density	Heterogeneous (↓ and slightly ↑)	Mostly ↑ with severely ↓ layers and ↓ septations	Mostly ↑ with severely ↓ layers and ↓ septations	Mass-like ↓	+	+	0	0
19	Mixed density	Heterogeneous, mainly slightly ↑	Mostly ↑ with severely ↓ layers and ↓ septations	Mostly ↑ with severely ↓ layers and ↓ septations	Mass-like ↓	+	+	0	0
20	Mixed density	Heterogeneous (most slightly ↑, severely ↑ occipital)	Mostly ↑ with severely ↓ layers and ↓ septations	Mostly ↑ with severely ↓ layers and ↓ septations	Mass-like ↓	+	+	0	+
21	Mixed density	Mostly ↔ with patchy ↑ parts	Homogeneously ↑ with ↓ septations	Homogeneously ↑ with ↓ septations	Linear ↓	+	+	+	+
22	Mixed density	Mostly ↑ with ↔ to ↓ layers and ↓ septations	Mostly ↑ with severely ↓ layers and ↓ septations	Mostly ↑ with severely ↓ layers and ↓ septations	Mass-like ↓	+	+	0	0
23	Mixed density	Mostly ↑ with small patchy ↓ parts and ↓ septations	Mostly ↑ with ↓ layers and ↓ septations	Mostly ↑ with ↓ layers and ↓ septations	Mass-like ↓	+	+	+	+
24	Isodense	Slightly ↑ with ↓ septations	Homogeneously ↑ with ↓ septations	Homogeneously ↑ with septations	Linear ↓	+	+	+	+
25	Mixed density	Homogeneously ↔ to slightly ↑, with two ↑ patches	Mostly ↑ with ↓ layers and ↓ septations	Mostly ↑ with ↓ layers and ↓ septations	Mass-like ↓	+	+	0	+
26	Mixed density	Mostly ↑ with small patchy ↓ parts and ↓ septations	Mostly ↑ with ↓ septations	Mostly ↑ with ↓ septations	Mass-like ↓	+	+	+	0
27	Hypodense	Homogeneously ↑ with ↓ septations	Homogeneously ↑ with ↓ septations	Homogeneously severely ↑ with ↓ septations	Linear ↓	0	+	0	0

↓, *hypointense;* ↑, *hyperintense;* ↔, *isointense;* CE, *contrast enhanced;* 0, *absent;* +, *present; linear* ↓, *linear hypointensity along septations or outer membranes; mass-like* ↓, *hypointensity of parts or full area of hematoma*.

The most frequent pattern was mixed signal in both T1- and T2-weighted images (14 hematomas, 52%), followed by hematomas with hyperintense signal on T2w and hypointense signal on T1w (4 hematomas, 15%).

On FLAIR images, the hematoma signal was similar to T2w images in 26 of 27 hematomas (96%). Only one hematoma showed hyperintense signal on T2w but hypointense signal on FLAIR images.

Overall, 20 hematomas (74%) showed internal septations (Figures 1–**3**). These septations were well seen in almost all sequences and more easily distinguished on T2w images and CISS images.

On average, the inner membrane measured about 1 mm in thickness on CISS images.

Diffusion restriction was noticed in 9 hematomas (33%), in all of which cases it was observed in parts of the hematoma only. In 4 hematomas (15%), the diffusion restriction was either linear or represented small patchy lesions along the outer membrane and/or internal septations. In the other 5 hematomas, the diffusion lesions were rather in a form of larger spots within the hematoma. The hematomas did thus not show overall diffusion restriction in their whole area. The mean ADC value in these lesions was 560 × 10^−6^ mm^2^/s (Figure 2). In the other hematomas with no diffusion restriction, the signal intensity on DWI varied between severe hypointensity in the areas with hypointensity in the T2^*^-GRE to mild hyperintensity in another few cases. However, in many cases, the hematoma had similar intensity compared to cerebrospinal fluid on DWI.

Hypointensity was noticed on T2^*^-GRE and SWI in 24 hematomas (89%) without any difference between both sequences. In 10 of these 24 cases, there were only linear hypointense signals along the inner or outer membrane and/or along the internal septations (Figures 1, 2). Interestingly, three hematomas (11%) did not show any hypointensity on T2^*^w images.

**Figure 4:**
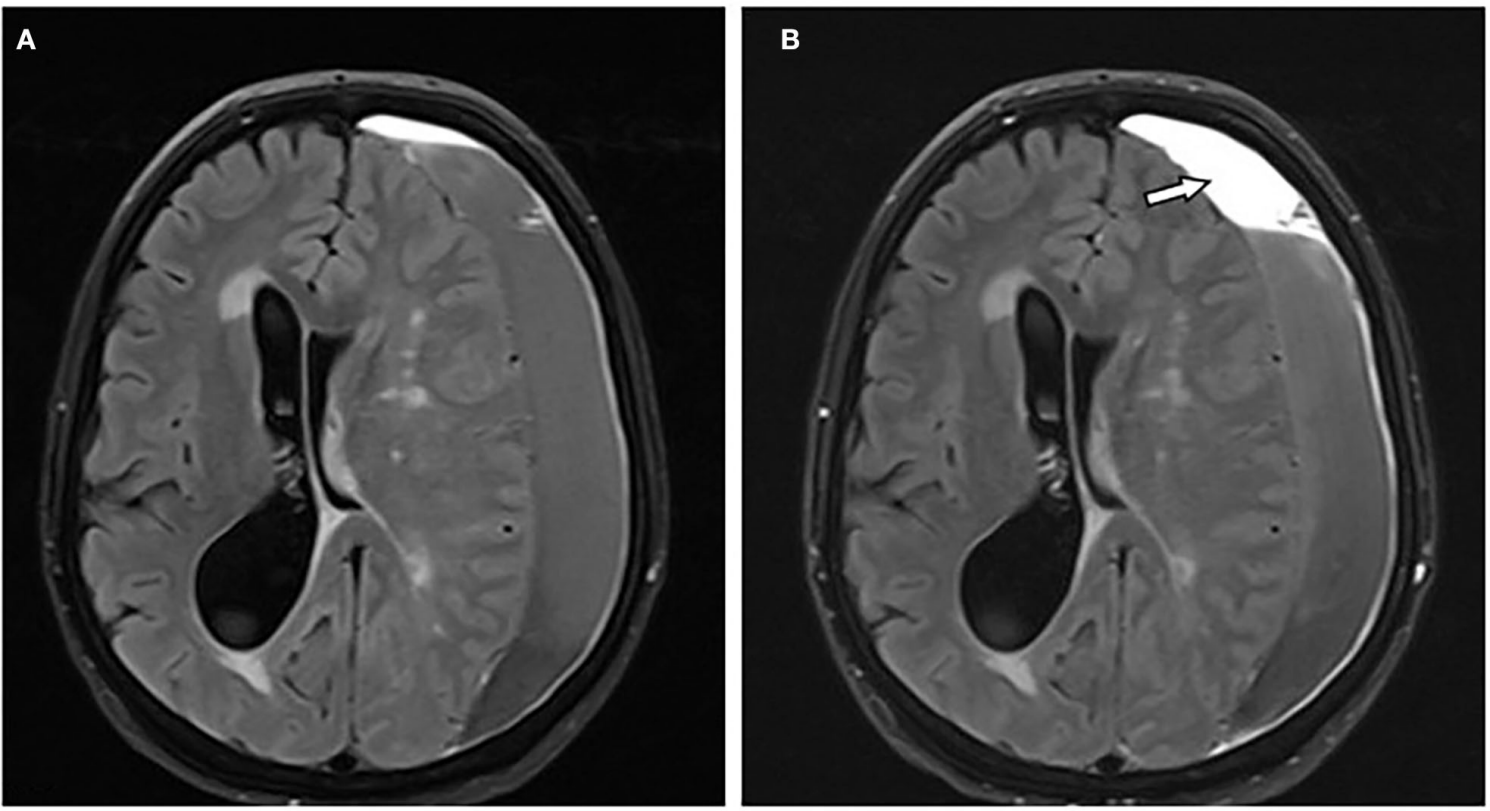
A 66-year-old female patient with chronic subdural hematoma (cSDH) over the left convexity. Axial T2-FLAIR images before **(A)** and after **(B)** contrast administration showing mass-like enhancement of the hematoma [**(B)**: arrow].

All hematomas (100%) showed linear contrast enhancement along the outer membrane adjacent to the skull (Figures 1, 3, 4). There was contrast enhancement along the inner membrane adjacent to the brain in only 10 cases (37%) (Figure 1). In these 10 cases, we noticed a triangular-shaped enhancement at the borders of the cSDH (Figures 1, 3). Enhancement of outer or inner membranes of the hematomas was observed on both T1w and FLAIR images, following contrast administration. In 17 hematomas (62%), we noticed a mass-like enhancement of the subdural hematoma (Figure 4). Interestingly, this mass-like enhancement was observed on 17 hematomas on FLAIR images but only in 4 hematomas on T1w images. This phenomenon was noticed mainly in hematomas without restricted diffusion. Only 4 of these hematomas (15%) had patchy diffusion restriction; and to our surprise, mass-like contrast enhancement was only outside these diffusion-restricted areas.

Regarding imaging characteristics on CT, most hematomas (*n* = 16, 64%) had mixed density on CT with hyperdense components, 7 hematomas (28%) were isodense, and only two hematomas (8%) were isodense.

## Discussion

Our main findings were that the signal characteristics of cSDH on T1- and T2-weighted images were very heterogeneous and that the most frequent pattern in our study was a mixed signal in both sequences. The majority of hematomas (74%) had internal septations.

To our surprise, all hematomas showed contrast enhancement. More specifically, contrast enhancement along the outer membrane adjacent to the cranium was noticed in all hematomas. There was also contrast enhancement along the inner membrane adjacent to the brain in approximately one-third of cases (37%). In approximately two-third of the cSDH (62%), there was a mass-like enhancement of the hematoma. If present, the latter phenomenon was always observed on FLAIR images but only rarely on T1w images. Most hematomas (89%) had hypointensity on T2^*^-GRE and/or SWI. Diffusion-restriction was detected in approximately one-third of the hematomas (33%). These imaging findings represent hallmarks of cSDH pathophysiology.

Anatomically, subdural hemorrhages are not actually subdural, but located in a newly formed intradural space within the innermost layer of the dura, the so-called “dural border zone” (14, 15). This dural border zone consists of fibroblasts and collagen and differs pathophysiologically from the outer layer of the dura (16). Hence, the inner membrane of a subdural hematoma consists of the dural border zone adjacent to the arachnoid barrier cells, whereas the outer membrane consists mainly of the outer layer of the dura (meningeal dura) (14). Complex processes like angiogenesis, fibrinolysis, and inflammation promote the maturation of subdural hematomas and lead to the proliferation of the outer and inner membranes, which consists of vascular granulation tissue and also internal septa (17–19). This active/inflammatory process originates from the outer layer of the dura, which explains why there was contrast enhancement along the outer layer of all cSDH in our study. Hence, contrast enhancement in the outer membrane of cSDH is not an expression of vascular congestion that can also occur after mild traumatic brain injury, but rather an expression of an inflammatory process (20). This inflammatory process, which is assumed to be a motor for hematoma growth, expands from the outer membrane to the inner membrane *via* inflammatory cells that pass through the border between the outer and inner membrane (19). In these cases, the transition zone between the inner and outer capsule of the hematoma is thickened and triangular shaped, with a characteristic spandrel-like appearance (6, 21). Hence, contrast enhancement along the inner membrane, which contains mostly collagen and fibroblasts and is therefore rather inactive, is less frequent. This pattern with contrast enhancement along the outer membrane, triangular-shaped contrast enhancement at the borders of the cSDH, and infrequent enhancement of the inner membrane helps distinguish cSDH from empyema in the subdural space, which usually has a marked enhancement on both sides of the empyema (22, 23).

In addition, it is important to mention that MRI imaging enables us to evaluate the thickness of the internal membrane, which may be important for surgical treatment planning according to recent studies (24).

The inflammatory processes of the outer membrane do not only expand throughout the membranes, but supposedly also through the hematoma, resulting in newly formed septations within the hematoma (19). Septations were found in almost three-quarters of our cSDH. In fact, septations are a frequent finding that can also be an expression of resorption and organization of hematomas along their peripheral zones (10, 25, 26).

Remarkably, with 62% of the cases, more than half of cSDH showed mass-like contrast enhancement, a finding that was also reported in 3 of 6 cases by Spreer et al. and in 2 case reports by Nagasaka et al. (6, 21). Interestingly, ubiquitous mass-like contrast enhancement is not a typical finding of parenchymal hematomas, which rather show circumferential enhancement during organization and resorption (27). It is possible that mass-like contrast enhancement in cSDH is rather the result of contrast leakage *via* the inflammatory membranes into non-solid hematomas. This is supported by a previous study by Mori et al. who showed that subdural effusions and more recent subdural hematomas enhanced faster than older cSDHs (28). This is also in line with our own observation that mass-like contrast enhancement was never found within diffusion-restricted and, hence, presumably solid parts of hematomas. Possible implications of this are that ubiquitous contrast enhancement as a surrogate for contrast leakage into non-solid hematomas could serve both as an indicator for potential hematoma growth and as a marker for successful endovascular embolization, i.e., an issue that is worth studying in future research (29).

With regard to hematoma growth and recurrence, Goto et al. described a high recurrence rate in cSDH with isointense and hypointense signals on T1-weighted images (30). The authors speculate that hematoma stability depends on the stage of the hematoma cycle with T1-hypointense hematomas representing acute and unstable bleedings with a higher risk for recurrent hemorrhage (30). As our study lacks longitudinal follow-up, we can neither prove nor disprove this hypothesis. In terms of MRI signal characteristics, cSDH behaves differently from parenchymal hematomas: It is well-known that the appearance of intracerebral hematomas on T1- and T2-weighted imaging depends on the imaging characteristics of the various blood degradation products (31, 32). A hematoma in the subdural space is expected to go through the same stages as parenchymal blood, only more slowly, due to greater oxygen tension in the subdural compartment (8, 33). However, the appearance of cSDH in our study was rather heterogeneous. There are multiple factors that explain the varied manifestations of cSDH: one of these factors is the recurrent hemorrhage in the hematoma cavity, resulting in the simultaneous appearance of blood degradation products of different ages. Beyond that, hemolysis within cSDH increases protein concentration which affects both T1- and T2-weighted intensity (9, 30, 34, 35).

In addition to the T1 and T2 signal, diffusion restriction on DWI can serve as an indicator of the state of coagulation of a hematoma, representing more solid parts (36). Consisting with this, diffusion restriction was not present in all hematomas, but rather found as small patchy areas in presumably solid parts of cSDH without contrast enhancement. This phenomenon was also reported in earlier studies and used to distinguish solid from liquid parts of cSDH. It was also reported that the presence of linear diffusion restriction along the outer membrane or the neomembrane in a cSDH may refer to new bleeding from this membrane (37, 38). In our study, we noticed a similar aspect of diffusion restriction in 15% of cSDH. The absence of diffusion restriction can help to distinguish cSDH from empyema, which is known to have ubiquitous diffusion restriction due to the high viscosity of the pus within the empyema (12, 13).

It is further of interest to notice that even though hemosiderin is supposed to be a hallmark of chronic hemorrhage, hypointensity on T2^*^-GRE and/or SWI was present in most but not all of our cSDH. A possible explanation for this is that, unlike in parenchymal hemorrhage, hemosiderin-laden macrophages that scavenge blood degradation products can exit the subdural compartment (8, 39). This is supported by our observation that the hemosiderin staining was sometimes only found adjacent to the dural surface and septa, which can be regarded as the areas with an exchange between hematoma and dural space. The presence of a hemosiderin staining in a subdural fluid collection may help us to differentiate cSDH from hygromas, which do not have such a hemosiderin staining.

## Limitations of the study

Our study has some limitations which need to be addressed. First of all, there are no serial MRI scans. As the cSDH changes over time, it would be very useful to see the changes in enhancement patterns that occur as time progresses. Second, our sample size is small in comparison with the high prevalence of this condition. However, the indication of CE MRI in this condition is still very limited in clinical practice, which has made finding a suitable cohort of patients very difficult. Another limitation is the retrospective nature of the study.

## Conclusion

Despite our relatively small sample size and the lack of longitudinal data, our study provides novel insights into the appearance of cSDH on MRI. Consistent contrast enhancement along the outer membrane, triangular-shaped contrast enhancement at the borders of the cSDH, and infrequent enhancement of the inner membrane may help to distinguish cSDH from other entities such as empyema and lymphoma. Mass-like enhancement may refer to non-solid hematomas and could be an indicator for hematoma growth and a possible surrogate for successful endovascular embolization. Partial DWI restriction of cSDH is possible but differs in appearance from the DWI restriction displayed by empyemas.

## Data availability statement

The original contributions presented in the study are included in the article/supplementary material, further inquiries can be directed to the corresponding author.

## Ethics statement

The protocol for this retrospective study was approved by the Independent Ethics Committee at the RWTH Aachen Faculty of Medicine. As this was a retrospective study, written informed consent was not required from patients. Written informed consent was not obtained from the individual(s) for the publication of any potentially identifiable images or data included in this article.

## Author contributions

All authors listed have made a substantial, direct, and intellectual contribution to the work and approved it for publication.

## Conflict of interest

The authors declare that the research was conducted in the absence of any commercial or financial relationships that could be construed as a potential conflict of interest.

## Publisher's note

All claims expressed in this article are solely those of the authors and do not necessarily represent those of their affiliated organizations, or those of the publisher, the editors and the reviewers. Any product that may be evaluated in this article, or claim that may be made by its manufacturer, is not guaranteed or endorsed by the publisher.
